# Mesenchymal stem cells and macrophages interact through IL-6 to promote inflammatory breast cancer in pre-clinical models

**DOI:** 10.18632/oncotarget.12694

**Published:** 2016-10-15

**Authors:** Adam R. Wolfe, Nicholaus J Trenton, Bisrat G. Debeb, Richard Larson, Brian Ruffell, Khoi Chu, Walter Hittelman, Michael Diehl, Jim M Reuben, Naoto T. Ueno, Wendy A. Woodward

**Affiliations:** ^1^ MD Anderson Morgan Welch Inflammatory Breast Cancer Research Program and Clinic, The University of Texas MD Anderson Cancer Center, Houston, TX, USA; ^2^ Department of Radiation Oncology, The University of Texas MD Anderson Cancer Center, Houston, TX, USA; ^3^ Breast Medical Oncology, The University of Texas MD Anderson Cancer Center, Houston, TX, USA; ^4^ Department of Experimental Therapeutics, The University of Texas MD Anderson Cancer Center, Houston, TX, USA; ^5^ Department of Bioengineering, Rice University, Houston, TX, USA; ^6^ Department of Immunology, H. Lee Moffitt Cancer Center and Research Institute, Tampa, FL, USA

**Keywords:** inflammatory breast cancer, macrophages, mesenchymal stem cells, IL-6, statins

## Abstract

Inflammatory breast cancer (IBC) is a unique and deadly disease with unknown drivers. We hypothesized the inflammatory environment contributes to the IBC phenotype. We used an *in vitro* co-culture system to investigate interactions between normal and polarized macrophages (RAW 264.7 cell line), bone-marrow derived mesenchymal stem cells (MSCs), and IBC cells (SUM 149 and MDA-IBC3). We used an *in vivo* model that reproduces the IBC phenotype by co-injecting IBC cells with MSCs into the mammary fat pad. Mice were then treated with a macrophage recruitment inhibitor, anti-CSF1. MSC and macrophages grown in co-culture produced higher levels of pro-tumor properties such as enhanced migration and elevated IL-6 secretion. IBC cells co-cultured with educated MSCs also displayed enhanced invasion and mammosphere formation and blocked by anti-IL-6 and statin treatment. The treatment of mice co-injected with IBC cells and MSCs with anti-CSF1 inhibited tumor associated macrophages and inhibited pSTAT3 expression in tumor cells. Anti-CSF1 treated mice also exhibited reduced tumor growth, skin invasion, and local recurrence. Herein we demonstrate reciprocal tumor interactions through IL-6 with cells found in the IBC microenvironment. Our results suggest IL-6 is a mediator of these tumor promoting influences and is important for the IBC induced migration of MSCs.

## INTRODUCTION

Inflammatory breast cancer is a deadly variant of breast cancer that presents with skin changes on the breast and spreads rapidly as a local, diffuse, “brush-fire” through the breast and adjacent tissue. These signs are caused in part by blockage of lymphatics and skin invasion, not by classic inflammation [1]. To date, studies comparing IBC and non-IBC tumor cells have identified more similarities than differences. Our preliminary studies of non-tumor containing breast tissue at least 5 cm away from IBC tumor cells (normal adjacent tissue or the “field”) have identified interesting differences, however. One such difference is that that the number of CD 68^+^ cells identified by a pathologist as histologically macrophages was significantly elevated in IBC normal adjacent tissues compared to non-IBC [2, unpublished data]. We hypothesized that the normal breast phenotype including, macrophage infiltration and resultant interactions with MSC, perhaps prior to tumor initiation, may contribute to the unusual presentation of IBC.

Macrophages are plastic cells broadly and simplistically characterized on a spectrum from Th1 stimulated pro-inflammatory to Th2 stimulated, anti-inflammatory [3]. Tumor associated macrophages are prognostic in non-IBC and have been shown *in vivo* to promote lymphatic emboli, the hallmark of IBC [4]. The state and plasticity of macrophages (tumor associated or normal adjacent) in IBC models or patients and models has not been examined, nor has their interaction with other cell types in the IBC stroma. Such knowledge is important because these cells are potentially mediators of field effects that lead to the clinical characteristics of IBC, and therefore may be targets for treatment of IBC.

We have shown that mammosphere cultured MSCs promote tumor stem cells and *in vivo* growth [5], and Liu et al have shown that ALDH1+ MSCs do the same [6]. MSCs have also been demonstrated to mediate macrophage polarization and function [7]. Yet, the impact of macrophages on MSCs has largely been overlooked.

We hypothesized that pre-malignant macrophage-educated MSCs in the breast mediate the IBC phenotype including diffuse migration, skin involvement, and treatment resistance. This hypothesis is based on our data demonstrating obvious macrophage infiltrates specifically in IBC normal adjacent tissue, unique spatial distribution of stem cells in the adjacent normal tissue of IBC, *in vitro* and *in vivo* data demonstrating MSCs promote stem cell surrogates and skin invasion of IBC, as well as published studies highlighting the role of macrophage-educated MSCs in supporting the hematopoietic stem cell niche. Herein we demonstrate macrophages are essential for the MSC promotion of skin invasion in the SUM149 model and that these interactions are dependent on IL-6 signaling.

## RESULTS

### Characterizing the interactions between MSCs and macrophages in a co-culture system

**Figure 1:**
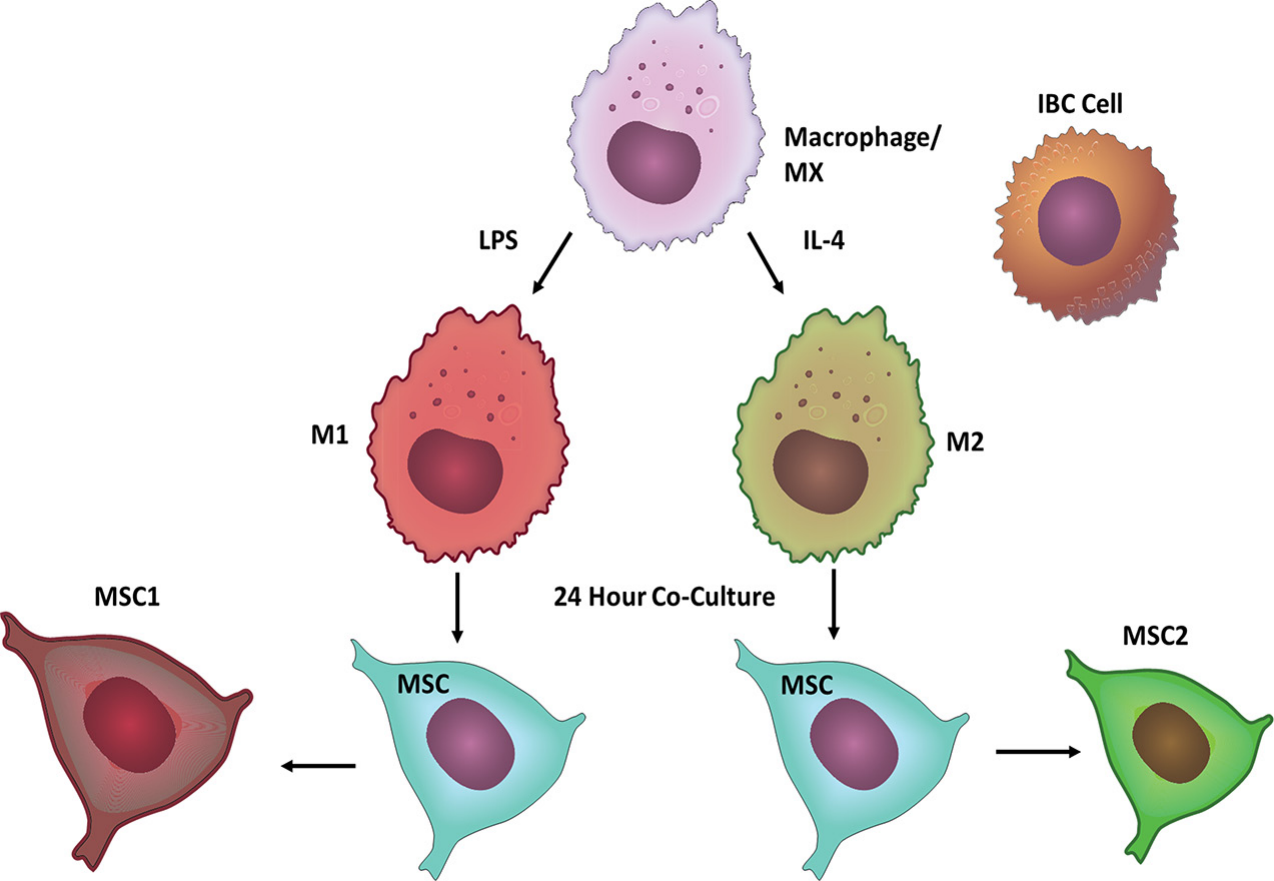
Model of Co-Culture System Macrophages were polarized with either LPS or IL-4 to become M1 or M2 macrophages respectively. Using a Boyden chamber MSCs were seeded with M1 or M2 macrophages for 24 hours to become either MSC1s or MSC2s. MSC1s or MSC2s were then co-cultured in Boyden chambers with IBC cells for 24 hours. Following co-culture MSCs and IBC cells were analyzed for migration, invasion, and mammosphere formation.

**Figure 2:**
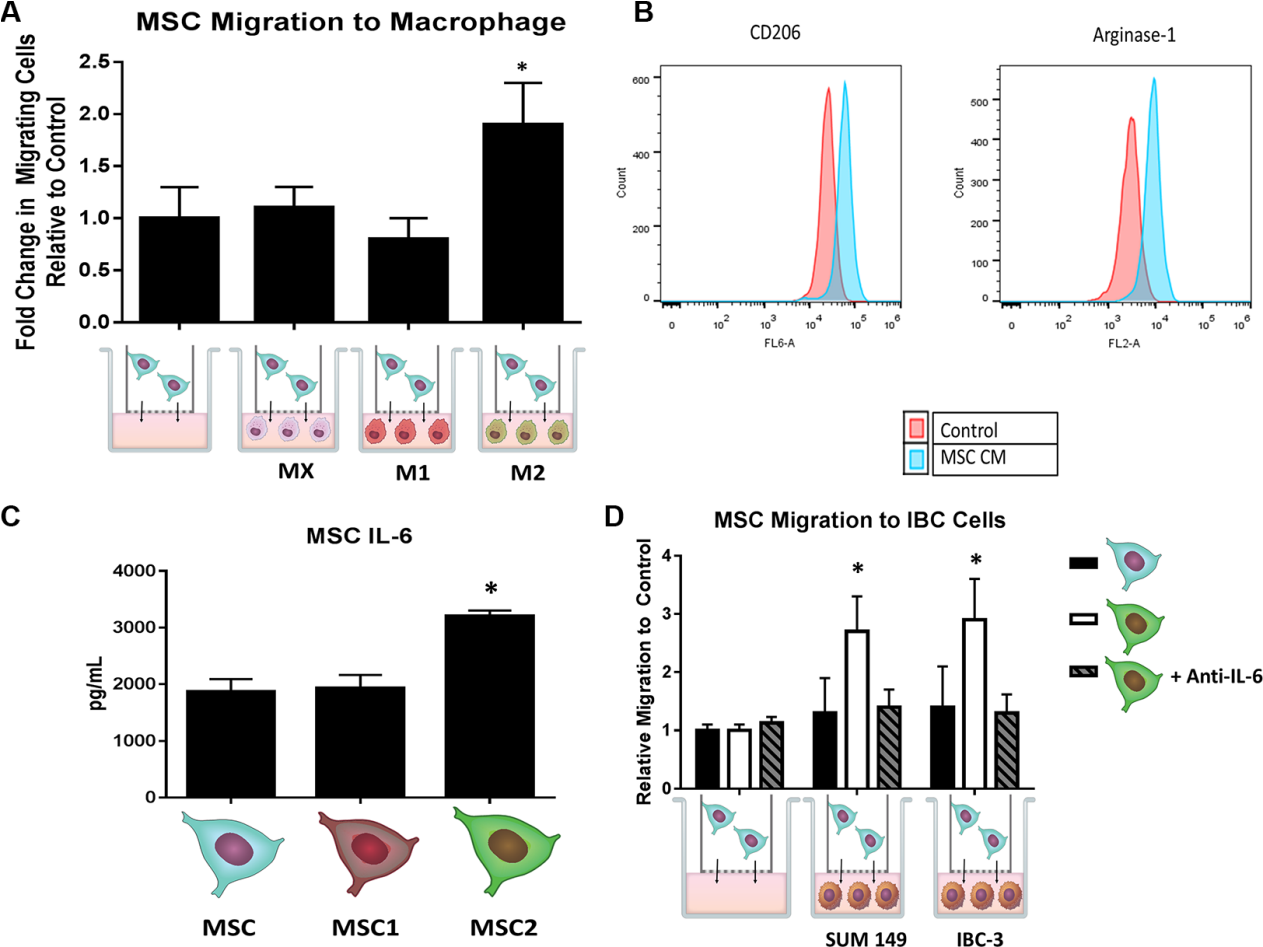
MSCs and macrophages cross-talk through IL-6 Following co-culture of with either un-polarized (M0), LPS induced (M1), or IL-4 induced (M2), and MSCs for 24 hours, (**A**) the number of MSCs that passed through the transwell membrane after 24 hours was counted. Significant differences are shown as follows: *P < 0.05 for unpaired 2-tailed Student's *t*-test (*n* = 3). (**B**) Macrophages were grown in the presence of MSC conditioned media (CM) for 24 hours and then stained with either anti-CD206 or anti-arginase-1 antibodies and submitted for flow cytometry analysis. (**C**) Following 24 hours of co-culture with macrophages and MSCs, supernatant was collected for IL-6 ELISA. (**D**) MSC or MSC2s were co-cultured with SUM149 and IBC-3 cells for 24 hours with or without anti-IL6 antibodies. The number of MSCs that passed through the transwell membrane after 24 hours was counted. Significant differences are shown as follows: *P < 0.05 for unpaired 2-tailed Student's *t*-test (*n* = 3).

To characterize the effect on MSC activation of macrophages, we cultured RAW 264.7 mouse macrophage cells with human-derived MSC cells in an *in vitro* transwell co-culture assay (Figure 1). The transwell setting allowed us to investigate the effect of soluble mediators between different cell types since direct cell contact of these cells was inhibited by a polyethylene membrane. The MSCs co-cultured with M2 macrophages migrated at an almost 2-fold increase on average compared to MSCs grown with serum alone, M0, or M1 macrophages (*p* < 0.05) (Figure 2A). Next, we examined the impact of MSCs on macrophage polarization. Un-stimulated macrophage cells were cultured *in vitro* for 24 hours in regular RAW media or MSC conditioned media (CM) and then collected for flow cytometry analysis for CD206 and Arginase-1, markers for M2 phenotype. MSC CM increased the percentage of CD206 and Arginase-1 compared with control macrophages (Figure 2B). We next performed a luminex bead-based multiplex cytokine assay to evaluate the cytokine profile of MSCs. The levels of IL-6 in the MSC media was several fold higher than all the other cytokines assessed (Supplementary Table S1). Furthermore, the MSC2-educated cells secreted 1.6 fold more IL-6 compared to MSCs or MSC1 educated cells (*p* < 0.05) (Figure 2C). To investigate how MSCs co-cultured with M2 macrophages impact MSC migration, we took MSC controls or MSC2-educated cells and transferred them to a co-culture system with two IBC cell lines (SUM 149 or IBC3) for 24 hours. We found MSC2 educated cells co-cultured with either of the IBC cell lines exhibit greater migration towards tumor cells compared with uneducated control MSCs (*p* <.05). This observed effect could be reversed with the addition of an anti-IL-6 neutralizing antibody during the 24 hour co-culture (Figure 2D).

### Crosstalk between MSC2-educated cells and IBC cells increases IBC invasion and self-renewal through IL-6

**Figure 3:**
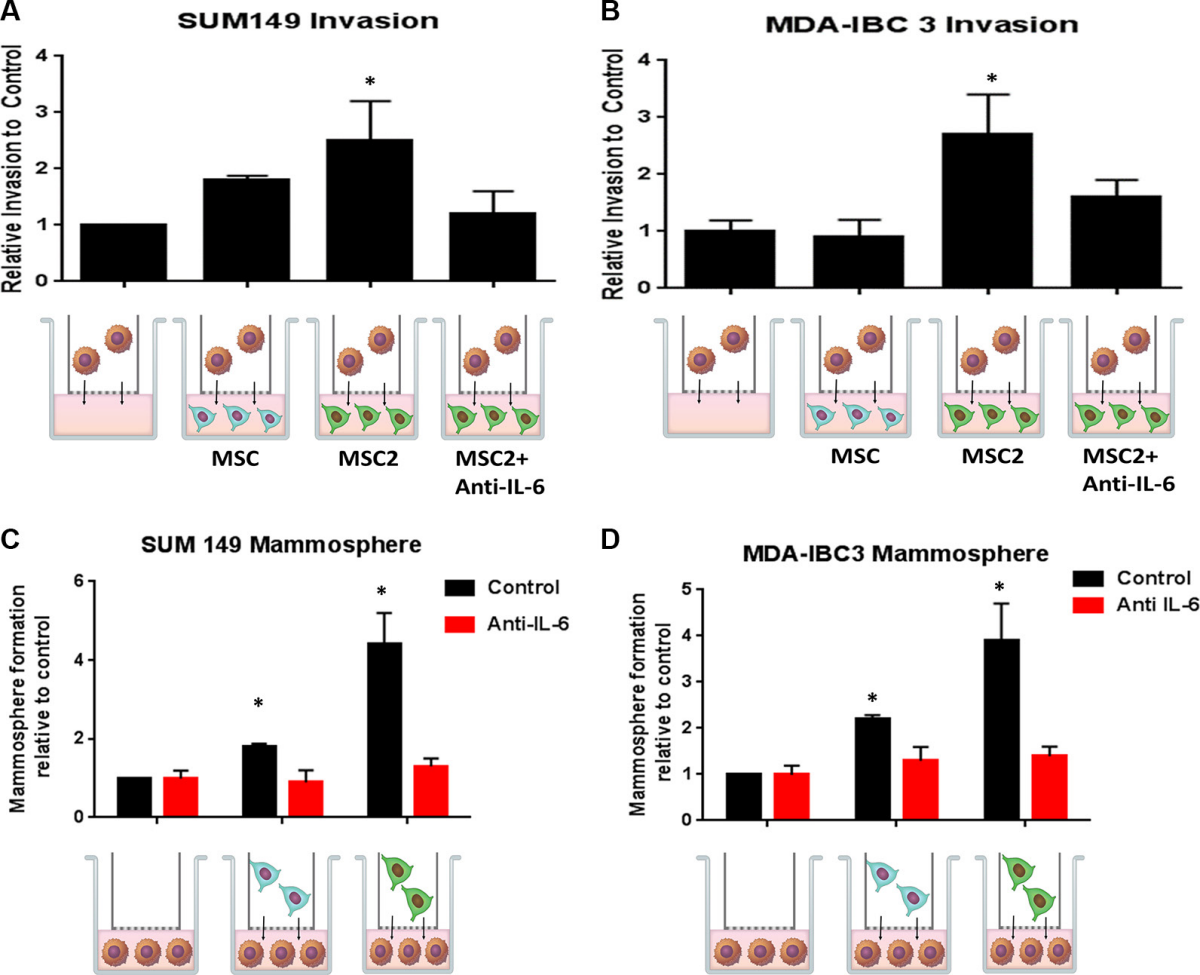
Crosstalk between educated MSCs and IBC cells increases IBC invasion and self-renewal through IL-6 Following 24 hours of co-culture with M2 macrophages, the “educated” MSC2s or parental MSCs were co-cultured with (**A**) SUM149 or (**B**) MDA-IBC3 for 24 hours with or without anti-IL6 antibodies. The number of IBC cells that invaded through the basement membrane transwell after 24 hours was counted. Significant differences are shown as follows: *P < 0.05 for unpaired 2-tailed Student's *t*-test (*n* = 3). (**C**) SUM149 or (**D**) MDA-IBC3 cells were co-cultured with MSC or MSC2s for 24 hours with or without anti-IL6 antibodies. IBC cells were then seeded in self-renewal mammosphre media promoting suspension culture conditions. Significant differences are shown as follows: *P < 0.05 for unpaired 2-tailed Student's *t*-test (*n* = 3).

We next investigated how the IBC cells would respond following 24 hours co-culture with MSC2s. The IBC cells co-cultured with MSC2s had a significant higher number of invading cells compared with co-culture with serum alone or MSCs (Figure 3A). IBC3 cells grown in co-culture with MSC2s had a 3 fold increase in invasion compared with serum alone (Figure 3B). The increase in invasion of M2 educated-MSCs was inhibited with the addition of the anti-IL-6 antibodies during the co-culture (Figure 3A, 3B). We next assessed IBC mammosphere formation following co-culture. Co-culture with both MSCs and M2-educated MSC2s increased SUM149 and IBC3 sphere formation efficiency. However, MSC2s increased sphere formation more strongly compared with control MSCs (Figure 3C, 3D). Once again this effect on mammosphere formation was blocked with anti-IL6 antibodies (Figure 3C, 3D).

### Simvastatin inhibits MSC IL-6 secretion and inhibits the effects of M2 educated-MSCs on IBC invasion and self-renewal

**Figure 4:**
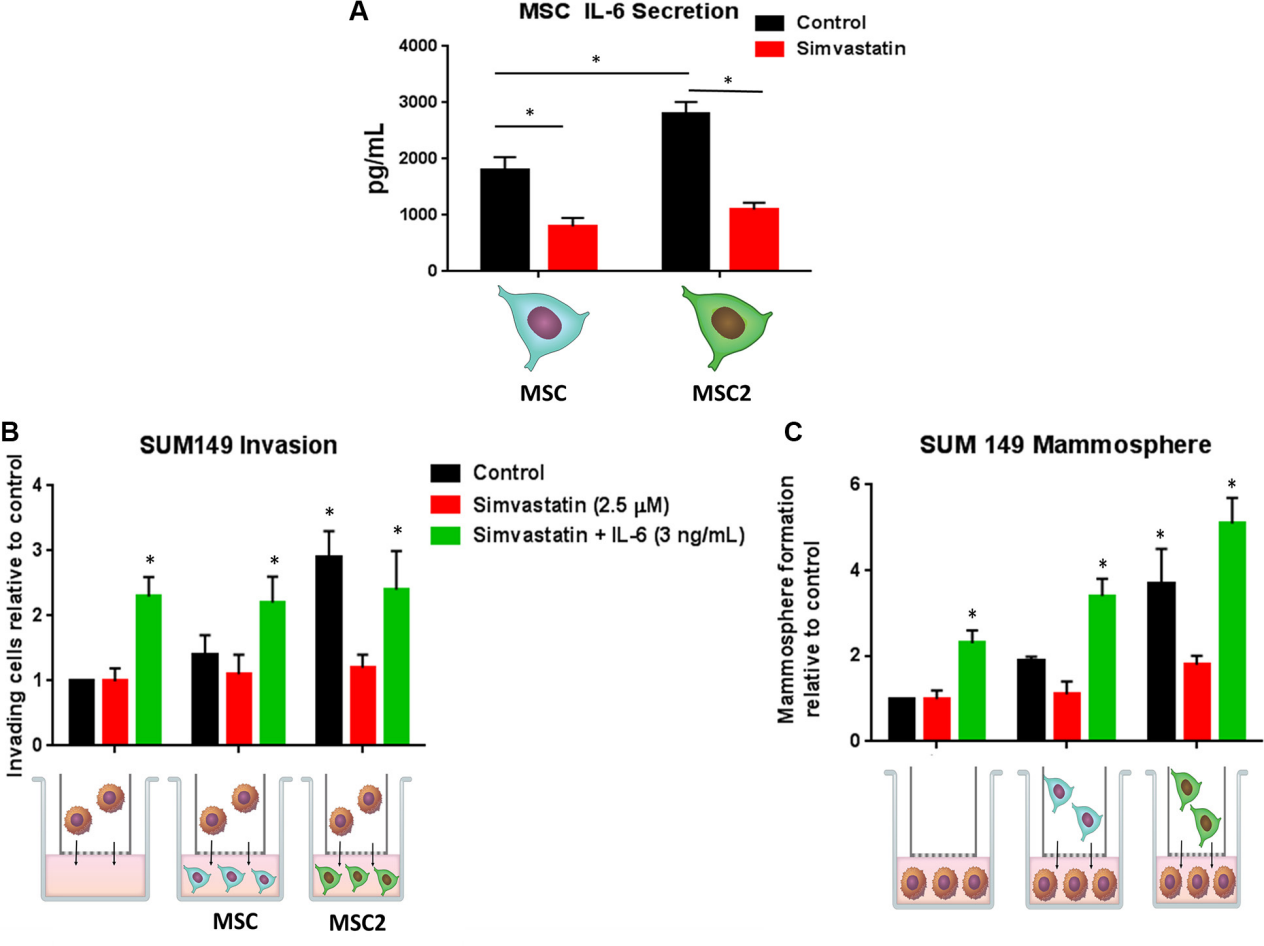
Simvastatin blocks IL-6 secretion and inhibits the effects of M2 educated MSCs on IBC invasion and self-renewal (**A**) MSC or MSC2s were treated with DMSO (control) or simvastatin for 4 hours and the supernatant was submitted for IL-6 ELISA. MSCs and MSC2s were either pre-treated with simvastatin or simvastatin plus IL-6 for 4 hours then co-cultured with SUM149 cells for 24 hours. (**B**) Following co-culture the number of SUM149 cells that invaded through the basement membrane transwell after 24 hours was counted. (**C**) SUM149 cells were seeded in self-renewal promoting suspension culture conditions. Significant differences are shown as follows: *P < 0.05 for unpaired 2-tailed Student's *t*-test (*n* = 3).

Studies have shown the cholesterol lowering drugs, statins, promote the pro-inflammatory macrophage subtypes and reduce the release of IL-6 in smooth muscular cells [8, 9]. We therefore tested the effects of simvastatin in our co-culture system. As seen before, MSC2s secreted more IL-6 compared with MSCs, while simvastatin reduced by over 2-fold the secretion of IL-6 in MSCs and MSC2s (Figure 4A). We next pre-treated MSCs or MSC2s with simvastatin or DMSO for 4 hours and then co-cultured the treated or untreated MSCs with IBC cells. As observed before, MSC2s significantly increased the number of mammospheres and invading cells of SUM 149 cells following 24 hour co-culture. The treatment of the MSC2s with simvastatin prior to co-culture with SUM149 cells inhibited both invasion and mammosphere formation of SUM 149 cells (4B, 4C). The addition of recombinant IL-6 reversed the above inhibition (Figure 4B, 4C). These results suggest simvastatin blocks MSC2 activation of IBC cells through reduction of IL-6.

### Inhibiting tumor associated macrophage recruitment in an *in vivo* IBC model delayed tumor formation and decreased skin invasion and local recurrence and decreased pSTAT3

**Figure 5:**
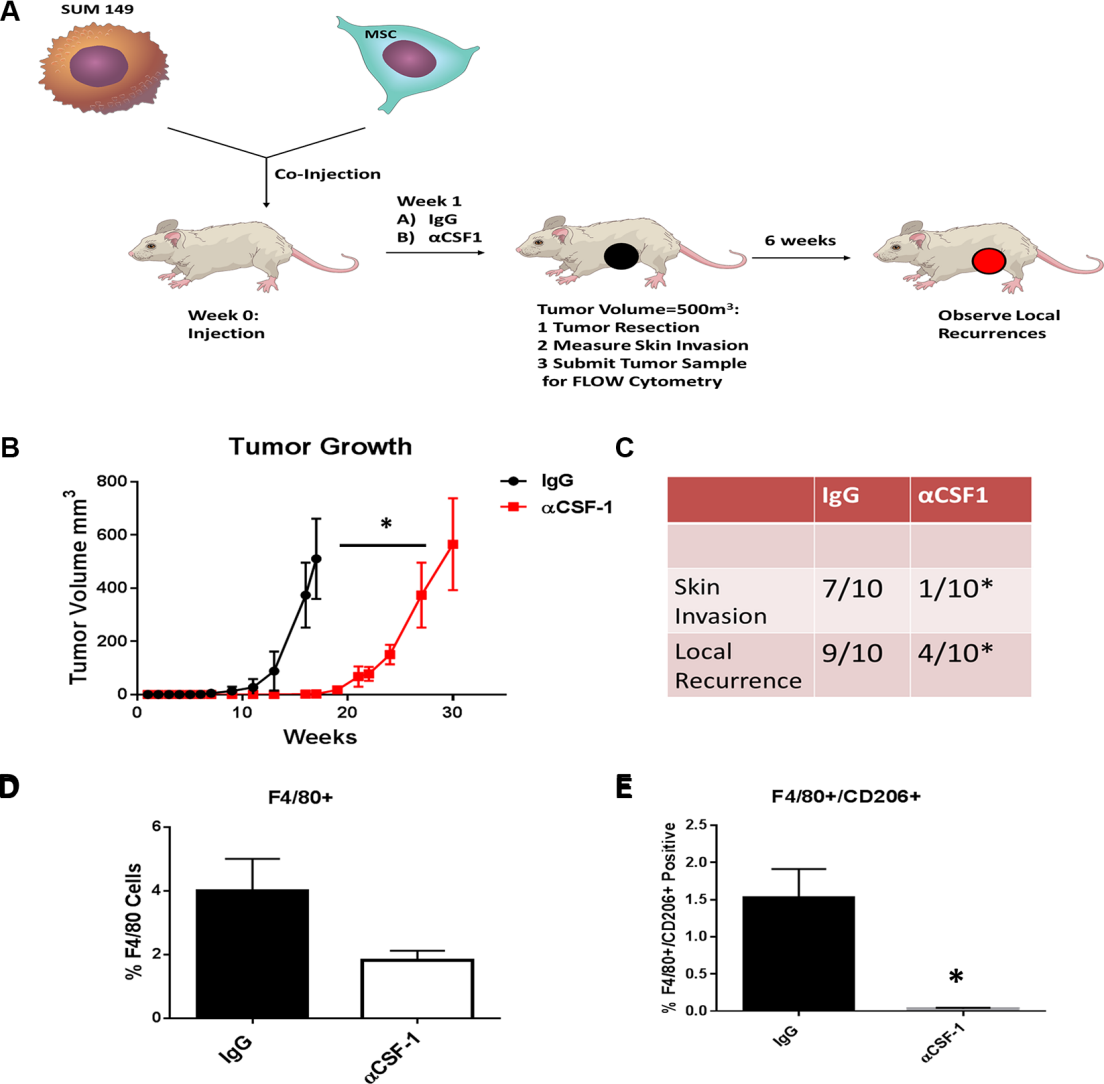
Inhibiting M2 macrophage recruitment in the *in vivo* IBC model inhibites IBC growth and skin invasion (**A**) Cell suspensions of SUM149 cells and human bone marrow derived MSCs were prepared from monolayer cultures. In both groups, SUM149 and MSCs (9:1 ratio) were mixed and then co-injected into the mammary cleared fat pad as previously described. 24 hours following co-injection of tumor cells and MSCs, we started treatment with either anti-CSF1 antibody or IgG intraperitoneally. We continued with injections of these antibodies weekly up until the week before resection of the tumor. (**B**) Weekly tumor measurements were made. (**C**) The presence of skin invasion and following tumor resection the presence of local recurrence was recorded. Tumors from both groups were resected and collected for digestion. Tumor samples were either stained with (**D**) anti-F4/80 antibodies or (**E**) both anti-F4/80 and anti-CD206 antibodies. The results of 3 replicates are shown by flow cytometry. Significant differences are shown as follows: *P < 0.05 for unpaired 2-tailed Student's *t*-test (*n* = 3).

Colony stimulating factor-1 (CSF1) is a key cytokine involved in recruitment and activation of tissue macrophages [10]. Denardo et al showed an antibody against CSF1 blocked the binding of CSF1 to its CSF1 receptor and inhibited tumor associated macrophages (TAM) within the breast tumor [11]. The co-injection of both SUM149 cells and human bone marrow derived MSCs injected into the mammary fat pad was previously shown to replicate the pathologically and phenotypical characteristics seen in human IBC patients [12]. A model of our *in vivo* experiment is represented in Figure 5A. Mice were co-injected with SUM149 and MSCs into the mammary cleared fat pad on day 0. On day 7 treatment with either anti-CSF1 antibody or IgG IP was started and continued weekly. Monitoring tumor growth we observed that tumor latency was significantly shorter in the IgG mice (68.3 days vs. 124.4 days, *P* <.001; Figure 5B). The time to reach tumor resection in the anti-CSF1 was slower than tumors in the IgG group (105.7 days vs. 209.3 days, *P* < 0.001; Figure 5B). At the time of resection we recorded whether or not the tumor involved the skin as described in methods. The group of mice treated with anti-CSF1 had significantly fewer mice with skin symptoms compared to the IgG group (7/10 IgG vs 1/10 anti-CSF1 *p* = 0.02). Further, following resection we monitored for local recurrence using live bioimaging to detect luciferase signal 6 weeks following surgery. The IgG group had significantly more events of local recurrence compared to the anti-CSF1 group (9/10 IgG vs 4/10 anti-CSF1 *p* = 0.05) (Figure 5C). The tumors were collected following resection and stained with macrophage specific antibodies F4/80 and the M2 marker CD206 and analyzed by flow cytometry. The anti-CSF1 group had a lower percentage of F4/80+ cells compared to the IgG group (Figure 5D). The anti-CSF1 group had a significantly lower percentage of double positive cells F4/80+ CD206+ cells, or TAMs, compared to the IgG group (*p* <.01) (Figure 5E). This suggests the anti-CSF1 antibody treatment *in vivo* reduced tumor macrophage recruitment and specifically blocked type 2 macrophage recruitment into the tumor and thus delaying tumor growth and skin invasion, hallmarks of IBC.

**Figure 6:**
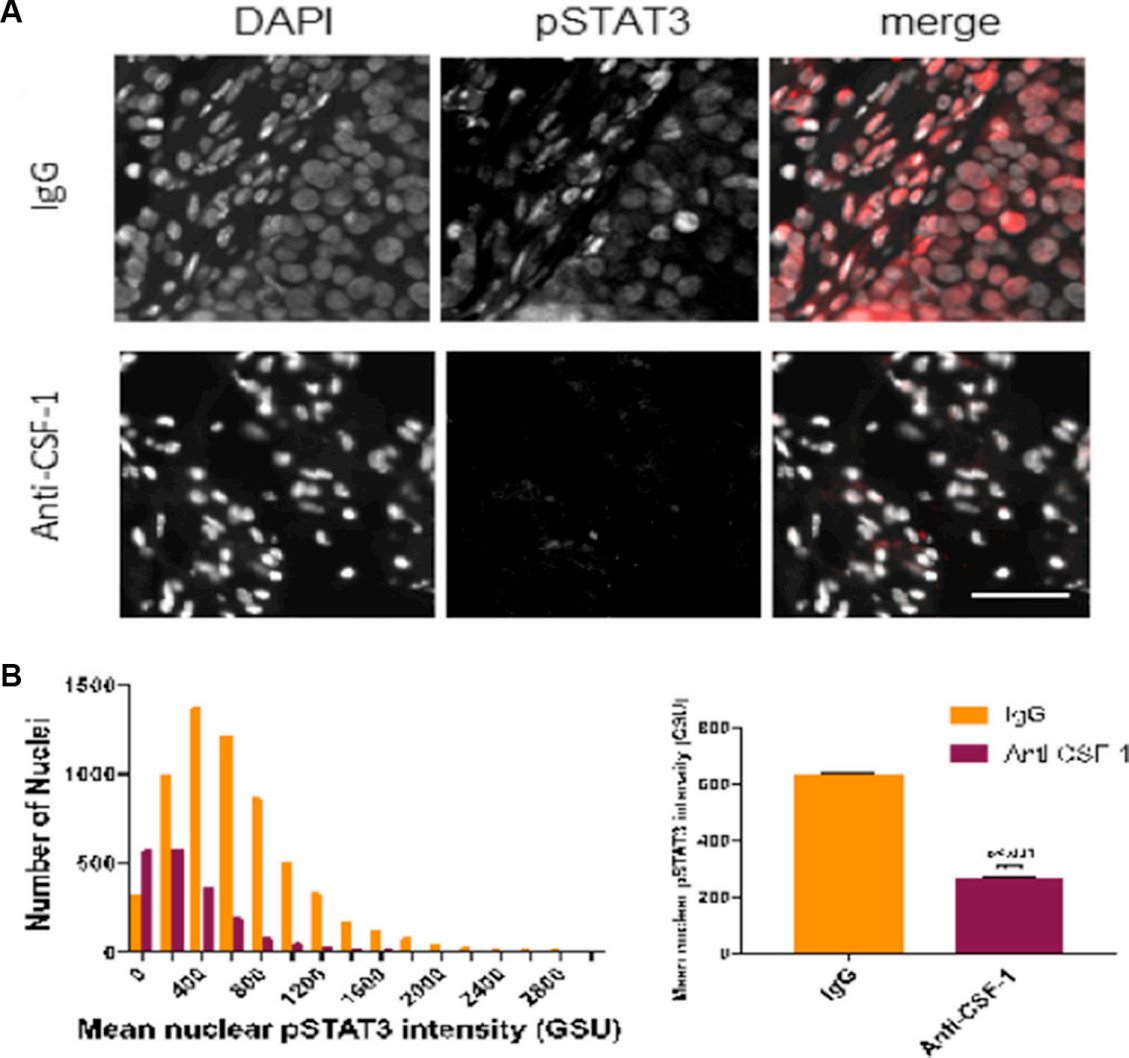
Inhibiting tumor associated macrophages reduces expression of pSTAT3 in tumor samples (A) Stitched fluorescent image (20X) of IgG tissue staining for DAPI (blue) and pSTAT3 (red) (**B**) Hematoxylin and eosin stain of IgG tumor tissue with arrow showing tumor invasion into the muscular skin layer. (**C**) Multiplex image (20×) of anti-CSF-1 tumor tissue staining for DAPI (blue) and pSTAT3 (red). (**D**) Hematoxylin and eosin stain of anti-CSF1 tumor tissue showing high degree of necrosis and undifferentiation. (**E**) Representative fluorescent image (20×) of IgG and anti-CSF1 mouse showing DAPI, pSTAT3, and merged images. (**F**) Left: representative histogram of mean nuclear pSTAT3 intensity (background-subtracted) between IgG and anti-CSF-1 tumor nuclei distributions (nnuclei = 3000 in a single tissue section analyzed for each sample). Right: mean nuclear pSTAT3 intensity (background-subtracted) across multiple mice (nIgG = 2 mice, nanti-CSF1 = 3 mice; 3 sections per mouse analyzed).

The binding of IL-6 to its receptor gp130 activates JAK2 which goes on to activate several downstream tumor promoters such as STAT3. Phosphorylated STAT3 (pSTAT3) activates cell survival factors and promoters of cell proliferation such as c-myc [13]. We next determined whether blocking macrophage recruitment in the above *in vivo* experiment reduced IL-6 secretion within the tumor environment by measuring pSTAT3 in the tumor samples. Examination of a cellular view, showed the IgG group had higher nuclear staining of pSTAT3 compared with the anti-CSF1 group (Figure 6A). The number of nuclei staining for pSTAT3 and the mean intensity of pSTAT3 staining was significantly higher in the IgG control group (Figure 6B).

## DISCUSSION

Macrophages are a major component of the innate immunity and are distributed throughout every tissue. After circulating monocytes differentiate into macrophages they invade into tissues and increase immune reactions to the response of injury [14]. In addition, macrophages remove cellular debris and clear dead or apoptotic cells [15]. Macrophages can be polarized in the environment in response to different stimuli [16]. The two classes of polarized macrophages are classically activated (M1) alternatively-activated (M2) macrophages [17]. M1 and M2 secrete different cytokines, and M2 is known to secrete IL-10, TNF-α and IL-6 [18].

Upon addition of MSC CM to macrophages, we observed an increase in expression of cell surface marker CD206 and Arginase 1, markers of the M2 phenotype. Additional studies by Ortiz et al. showed MSC-conditioned media inhibits the capacity of RAW- 264.7 cells activated by silica or LPS to secrete TNFα [19], and Nemeth et al. investigated the effect of mouse BM-derived MSCs in a murine model of septicemia and showed LPS-stimulated macrophages produced more IL-10 when co-cultured with BM-derived MSCs [20]. In our study MSCs co-cultured with M2 macrophages secreted higher levels of IL-6. We speculate that the crosstalk between MSCs and M2 macrophages is a driver of the IBC phenotype. In fact, these MSC2s enhanced IBC cell invasion and mammosphere formation. IL-6 signaling in tumor cells increases tumor growth by promoting tumor invasiveness, metastasis and angiogenesis [21]. Secretion of IL-6 leads to recruitment of metastatic cells out of the circulation to the primary tumor sites [22].

In addition to the effects on the tumor itself, recent studies show IL-6 controls tumor growth by activating the normal stromal cells located in the tumor microenvironment. For example, STAT3 activation initiated by IL-6 in tumor-associated endothelial cells, TAM, and MSCs induces their ability to express VEGF and bFGF in a feed-forward loop that positively regulates angiogenesis within tumor tissues [23]. Recent evidence suggests that MSCs coordinate with tumor cells that triggers increased IL-6 production, which correlates with accumulation of MSCs [24].

In an *in vivo* study, the combined treatment of MMTV-PyMT mice with paclitaxel and anti-CSF1 antibody slowed primary tumor development, reduced development of high-grade carcinomas, and decreased pulmonary metastasis by 85% and increased CD4+ and CD8+ T-cell infiltration in primary tumors. CSF1 receptor blockade by PLX3397 depleted TAMs, but not neutrophils [25]. Similarly in our *in vivo* experiment, CSF1 inhibition delayed SUM149 tumor initiation and reduced the percentage of TAMs (F4/80^+^/CD206^+^ cells). Importantly since IBC is characterized by rapid skin involvement, the reduction we saw in MSC induced skin invasion is noteworthy. These results suggest that the induction of the IBC phenotype with the addition of MSC cells is mediated by the host's macrophage infiltration, specifically M2 macrophages. Furthermore, pSTAT3 expression was inhibited by anti-CSF1 treatment suggesting that the inhibition of macrophage recruitment reduces the IL-6 signaling between MSCs and IBC cells. These data provide additional supportive evidence for a new clinical trial studying ruxolitinib (JAK inhibitor) in IBC (TBRCR trial # NCT02041429). Future studies are warranted looking drugs that can specifically target M2 type macrophages in patients with IBC. Our preliminary data suggest statins would be a well-tolerated FDA approved drug to investigate.

## MATERIALS AND METHODS

### Cell culture

The IBC cell line SUM149 was obtained from Asterand (Detroit, MI, USA) and cultured in Ham's F-12 media supplemented with 10% fetal bovine serum (FBS), 1 mg/mL hydrocortisone, 5 mg/mL insulin, and 1% antibiotic-antimycotic. MDA-IBC3 was generated in our lab and was described previously (Lacerda et al., PLoS One 2010) and was cultured in Ham's F-12 media supplemented with 10% fetal bovine serum (FBS), 1 μg/ml hydrocortisone, 5 μg/ml insulin and 1% antibiotic-antimycotic.

Human-derived bone marrow MSCs were obtained from EMD Millipore (Billerica, MA, USA) (Part #SCC034, Lot N61710996) and cultured in alpha minimum essential medium (αMEM) supplemented with 20% FBS and 1% penicillin/streptomycin/glutamine. The mouse macrophage cell line RAW 264.7 was purchased from ATCC and maintained in Dulbecco's Modified Eagle Medium (DMEM) supplemented with 10% FBS.

### Transwell co-culture assay

5 × 10^4^ of either breast cancer cells, macrophages, or MSCS were seeded in the lower compartment of 12 well transwell polyethylene terephthalate (PET) permeable supports in the 75 mm polycarbonate transwell inserts pore size 8 μm (Corning, Corning NY), respectively, and let to adhere overnight. The medium was replaced with serum free media one hour before adding 5 × 10^4^ of breast cancer cells, macrophages, or MSCs into the upper compartment of the transwell inserts. The co-cultures were incubated for 24 hours without medium change in a humidified chamber at 37°C. Following 24 hours, cells at the bottom of the co-culture assay were transferred for further experiments.

### Macrophage and MSC polarization

Macrophage polarization was obtained by removing the culture medium and culturing cells for an additional 24 h in DMEM media without FBS and 100 ng/ml LPS (for M1 polarization) or 20 ng/ml IL-4 and IL-13 (for M2 polarization). MSCs were plated at the bottom of the co-culture transwell system overnight. MSCs following 24 hours of co-culture with M1 or M2 macrophages are described as M1-educated or M2-educated furthermore respectively (Figure 1).

### Migration and invasion assays

Cells were assayed for their ability to migrate or invade using the cytoselect 24 well Cell Migration and Invasion Assay Kit (Cell Bio Labs, San Diego, CA). The Cell Migration portion of this kit uses polycarbonate membrane inserts (8 μm pore size) in a 24- well plate. The membrane serves as a barrier to discriminate migratory cells from non-migratory cells. 5.0 × 10^4^ of either MSCs, macrophages, or IBC cells were seeded in the top of the insert on the membrane. Serum free medium or one of the cell types was grown in the bottom of the transwell. After 24 hours of incubation at 37°C in a 5% CO2 atmosphere, the membranes containing the cells were fixed and stained with crystal violet. The lower surfaces of the membranes were photographed at × 100 magnification. Five random fields were photographed for each chamber to determine the migration.

The Cell Invasion Assay portion of this kit uses a 24-well plate containing polycarbonate membrane inserts (8 μm pore size); the upper surface of the insert membrane is coated with a uniform layer of dried basement membrane matrix solution. This basement membrane layer serves as a barrier to discriminate invasive cells from non-invasive cells. Invasive cells are able to degrade the matrix proteins in the layer, and ultimately pass through the pores of the polycarbonate membrane. Finally, the cells are removed from the top of the membrane and the invaded cells are stained and quantified.

### Sphere formation assay

5.0 × 10^4^ of IBC cells were plated on the bottom of the transwell assay overnight. 5.0 × 10^4^ MSCs or serum free medium were added to the insert. Following 24 hours of co-culture the IBC cells were transferred to standard mammosphere medium (serum-free, growth-factor-enriched medium) in low attachment plates at a concentration of 20,000 cells/mL, incubated for 7 days, and counted.

### IL-6 ELISA, IL-6, Anti-IL-6, and simvastatin treatment

IL-6 protein levels in culture supernatants were determined using commercial ELISA kits (eBioscience, San, Diego, CA). Recombinant human IL-6 and human IL-6 antibodies used to neutralize the binding of IL-6 to its receptor were purchased (R&D systems, Minneapolis, MN). IL-6 concentrations of 3 ng/mL and a concentration of 1 μg/mL of anti-IL-6 was used. Cells were exposed for 24 hours. Simvastatin (Sigma, St Louis, MO)) was dissolved in DMSO at a stock concentration of 5 mM and stored at −80°C, and a final concentration of 2.5 μM was used in this study. DMSO alone was used as a control.

### Flow cytometry

RAW macrophages were cultured in either RAW media described above or in conditioned media (CM) from MSCs for 24 hours. Following 24 hours 5 × 10^5^ of RAW cells were washed twice with PBS containing 1% goat serum and resuspended with 10 μL of anti-mouse CD206 Alexa Fluor 647, Arginase 1 PE (AbD Serotec), and 40 μL of the 1% serum PBS buffer. For tumor samples collected *in vivo,* cells were digested and washed in PBS containing 1% goat serum and resuspended in 10 μL of anti-mouse F4/80 PE (Ebioscience) and CD206. For both assays, samples were incubated for 20 minutes on ice, washed with 1% serum PBS buffer, and analyzed with the FACSCalibur. Cells with an isotype control for each antibody were used to set up the gate for analysis. The data were analyzed by the FlowJo software (version 8.8.6; TreeStar, Inc.).

### Co-injection of SUM149 cells with MSCs

SUM149 cells were injected into the cleared mammary fat pads of female immunocompromised SCID/Beige mice (3 to 5 weeks old), with MSCs (9:1 ratio), in a total of 2.5 × 10^5^ cells per injection. For co-injections, 2.5 × 10^4^ MSCs were premixed with GFP-labeled SUM149 cells and the mixture co-injected in the #4 (left side) mammary fat pad of the mice. Transplants were allowed to grow to 500 mm^3^ (monitored with caliper measurements) and were then resected in a survival surgery.

### Follow-up of tumor-skin involvement and metastasis development

Tumor-skin involvement was accessed visually during primary tumor growth (loss of fur at tumor site, redness and thickness of skin) and during tumor excision (tumors firmly connected with skin). The development of local recurrences was monitored by bioimaging the luciferase signal (IVIS Spectrum system (Caliper Life Sciences, Hopkinton, MA, USA)). Findings between groups were compared with Fisher's exact test.

### *In vivo* treatment with anti-CSF1

After co-injection of SUM149 cells and MSCs into the cleared mammary fat pads of female immunocompromised SCID/Beige mice as described above, mice were injected intraperitoneally (IP) with either mouse IgG or anti-mouse CSF1 antibodies (clone: 5A1, dose of 0.5 mg per injection) (BioXCell, West Lebanon, NH) started on day 7 following injection of cells and continued weekly up until the time of resection.

### Immunofluorescence

5 μm thick formalin-fixed, paraffin-embedded xenograft cross-sections harvested from mice treated with anti-CSF1 or IgG were stained with antibody against pSTAT3 (1:400, Cell Signaling Technologies Inc.) and secondary antibody AlexaFluor 647 (1:200, Invitrogen, Molecular Probes Inc.) and with DAPI (1:1000). Sections were deparaffinized by two sequential baths of xylene followed by 100% ethanol, 95% ethanol, 70% ethanol, and 30% ethanol. Antigen retrieval was performed using 1 mM EDTA (pH 8) in a 95°C steamer for 20 minutes, followed by permeabilization with 0.2% Triton-X 100. Sections were blocked for 30 minutes in blocking solution containing 10% normal donkey serum (Jackson ImmunoResearch), 1% (w/v) BSA (Jackson ImmunoResearch), and 0.02% Triton X 100 in PBS. Primary antibody was incubated in 10% blocking solution. Epi-fluorescent images of stained slides were captured on a Nikon Ti-Eclipse microscope equipped with Luca-R camera using a 20× (0.95 NA) objective and images were saved using Nikon NIS Elements software.

### Immunofluorescence quantification

After fluorescent images of DAPI and pSTAT3 were acquired, tumor cells were identified based on DAPI-stained nuclei using texture analysis through ImageJ image analysis software (NIH). Nuclei were thresholded into regions of interest (ROIs) using built-in auto thresholding techniques, and then background-subtracted pSTAT3 signal was overlaid on nuclear ROIs. Mean pSTAT3 intensity within each ROI was measured using ImageJ in at least 3000 nuclei per section (*n* = 2 mice for anti-CSF1 and *n* = 3 mice for IgG, with 3 blindly-chosen sections analyzed per mouse).

### Statistical analysis

Statistical analysis and graphing were performed using MATLAB 2016a (Mathworks Inc.) and Prism 7.0 (GraphPad Software Inc.). Mean pSTAT3 intensities were plotted on a histogram and representative distributions were generated for each condition (anti-CSF1 or IgG). A two-tailed *t*-test was used to analyze differences in mean pSTAT3 intensities between conditions (*p* <.05 statistical significance), with bar plot shown with mean ± SEM.

*T*-tests were performed using GraphPad Prism software, version 5.00 (GraphPad Software, San Diego, CA, www.graphpad.com) to determine statistically significant differences between groups. Differences were considered significant if *p* < 0.05. All graphs show mean ± SEM.

## CONCLUSIONS

We demonstrate reciprocal tumor interactions between normal cells in the IBC microenvironment. MSC and macrophages can influence each other to increase the tumor promoting influence of each on IBC cells. Our results suggest IL-6 a mediator of these tumor promoting influences and is important for the IBC induced migration of MSCs.

## SUPPLEMENTARY MATERIALS


