# Supplementary material for: Mesenchymal stem cells and macrophages interact through IL-6 to promote inflammatory breast cancer in pre-clinical models
Source: Oncotarget. 2016 Oct 15;7(50):82482–92. doi: 10.18632/oncotarget.12694 (PMC5347707; doi:10.18632/oncotarget.12694)
Supplement: Supplementary file 1 [file oncotarget-07-82482-s001.pdf]

Mesenchymal stem cells and macrophages interact through IL-6 to promote inflammatory breast cancer in pre-clinical models

Supplementary Materials

Supplementary Table S1:

	EGF	IFNg	IL-10	IL-12P70	IL-1a	IL-1b	IL-2	IL-3	IL-4	IL-5	IL-6	TNFa	VEGF
(pg/mL)	Obs Conc	Obs Conc	Obs Conc	Obs Conc	Obs Conc	Obs Conc	Obs Conc	Obs Conc	Obs Conc	Obs Conc	Obs Conc	Obs Conc	Obs Conc
MSC CM	0	8.47	0	0	0	0	0	5.01	0	0	1680.12	1.3	205.36
MSC CM	0	12.02	0	0	0	0	0	4.37	0	0	2064.71	1.71	215.18
